# One Chance to Get it Right: Exploring Perspectives and Experiences in Care Home Discharge Decision‐Making in the Acute Hospital

**DOI:** 10.1111/opn.70041

**Published:** 2025-09-05

**Authors:** Gemma Stevenson, Jennifer Kirsty Burton, Susan D. Shenkin, Juliet MacArthur, Brendan McCormack, Clare Halpenny, Sarah Rhynas

**Affiliations:** ^1^ Division of Nursing and Paramedic Science Queen Margaret University Musselburgh UK; ^2^ Academic Geriatric Medicine, School of Cardiovascular and Metabolic Health University of Glasgow Glasgow UK; ^3^ Department of Ageing and Health Usher Institute, The University of Edinburgh Edinburgh UK; ^4^ Advanced Care Research Centre Usher Institute, The University of Edinburgh Edinburgh UK; ^5^ NHS Lothian Edinburgh UK; ^6^ Queen's University Belfast Belfast UK; ^7^ Susan Wakil School of Nursing and Midwifery The University of Sydney Sydney New South Wales Australia; ^8^ Nursing Studies, School of Health in Social Science The University of Edinburgh Edinburgh UK

**Keywords:** acute hospital, admission, care home, decision‐making, discharge planning

## Abstract

**Background:**

Discharge from acute hospital to new care home is a complex and life changing process often involving several key stakeholders in decision‐making such as the older person, their significant person and members of the multidisciplinary team. There is limited research exploring the perspectives of these stakeholders, including factors that influence decision‐making and how this is communicated.

**Objective:**

This study explored how decisions are made to discharge older people directly from hospital to care home, considering the perspectives and experiences of those involved.

**Methods:**

A case study design was used to explore the experiences of six older people admitted to acute hospital from home for whom discharge to care home was planned. Six datasets were formed, each comprising semi‐structured interviews with the person, their significant person(s) (if applicable), multi‐disciplinary professionals and review of health and social‐work records. Datasets were analysed using an inductive thematic approach before cross‐dataset analysis.

**Results:**

Findings emphasised the complex and personal nature of decision‐making. The older person was often keen to talk about their decision. Significant people highlighted the complexity of balancing risk and care needs. However, the magnitude of the decision to older people and their significant persons appeared to go underacknowledged by professionals. The hospital context was significant as a location for decision‐making. Communication was integral to the experiences of those involved; however, uncertainty and lack of role clarity impacted this.

**Conclusions:**

This study offers new insights into the complexity of discharge to care home from hospital. This life‐changing decision requires greater recognition by professionals. Improved understanding of the process and well‐developed communication is central to enhancing the experience for those involved.

**Implications for Practice:**

The significance of this oftentimes final decision should not be underestimated. The findings indicate a clear need for interdisciplinary education about care home discharge, and the importance of professionals' availability and approachability throughout decision‐making. Professionals are encouraged to recognise a shared responsibility for the provision of information and guidance, and create opportunities for open and supportive conversations with older people and their families to explore the decision and discuss their feelings.

**Summary**

- What does this research add to existing knowledge in gerontology?
  ○ Discharge from the acute hospital to care home is a significant and life‐changing process and there is often only one chance to get it right.
  ○ Communication throughout decision‐making around care home transition from hospital is integral to the experiences of those involved; however, professional uncertainty and lack of role clarity can hinder these interactions.
  ○ The hospital context can be significant as a location for decision‐making; however, the magnitude of the decision is currently under‐recognised by professionals who observe many such transitions in their workplaces.
- What are the implications of this new knowledge for nursing care for and with older adults?
  ○ Interdisciplinary communication, improved education about care home discharge and professional role clarification are essential to support older people and their families throughout decision‐making.
  ○ There is a need to help prepare those professionals involved in decision‐making for the complexity of the situation and its significance to individuals and their families.
  ○ In light of increasing health and social care system pressures and continued need for care home admission from hospital, the preparedness, approachability and availability of professionals during the process are essential.
- How could the findings be used to influence policy or practice, education, research, and policy?
  ○ There is a need for interdisciplinary education about care home discharge, including the importance of open and supportive communication and the professionals' availability and approachability throughout decision‐making.
  ○ Future research could consider how simulated learning opportunities could be used to enhance nursing communication around hospital to care home transition.
  ○ Person‐centred care planning could highlight opportunities to discuss care home transition, raising awareness of the significance of this transition while prompting staff to engage in conversations about the person's future plans.
  ○ Research retrospectively exploring a person's experiences of care home transition, when they have settled in their care home, could provide insight into potential enhancements for support and preparation during acute hospital admission.

## 1 Introduction

The decision to move into a care home comes with significant implications for the person, associated with a sense of loss of family and community connections, familiar belongings and privacy (Scheible et al. 2019). Although challenges are recognised internationally, there has been a lack of research focusing on the perspectives of older people (Skudlik et al. 2023). Several factors influence the decision‐making process, including the role of support networks, cultural backgrounds, and the support provided within the hospital setting (Cole et al. 2018, 2021; Livingston et al. 2010; Samsi et al. 2019). Key aspects of this experience include information‐gathering, advocacy and system navigation, all of which can impact the transition phase (Hainstock et al. 2017).

When making this decision in hospital, concerns have been expressed that older people lack opportunities to be involved in discussions (Johnson et al. 2010; Lilleheie et al. 2019; Taylor and Donnelly 2006). Older people in hospital often experience poor health which can be compounded by feelings of stress, both for the person and their family (Johnson et al. 2010). Health crises are recognised as a catalyst for moving into care (Lam et al. 2025). Facilitating decision‐making in this context can be challenging. An additional stressor can be perceived, or real, pressure to discharge people from hospital, which can expedite the decision to move to a care home (Davies and Nolan 2003). Families describe feeling rushed to select a care home and staff report uncertainty about whose role it is to initiate care home conversations (Taylor and Donnelly 2006). Feeling supported and having thorough conversations about discharge planning are noted as important components of this process (Davies and Nolan 2003).

Older people who initiate the decision are reportedly more satisfied with their move to a care home than those who do not (O'Neill et al. 2022; Samsi et al. 2022; Kraun et al. 2023); and negative perspectives of the move are exacerbated when the decision has been made involuntarily (Samsi et al. 2019). However, much of the literature in this area focuses on older people moving into care homes from the community, rather than directly from hospital (Zhang et al. 2024). The increasing pressures to expedite discharges (Rojas‐Garcia et al. 2017) and a more complex and aging inpatient population globally (Naik et al. 2024) make the hospital a distinct context requiring specific research. Most people moving in to care homes in Scotland move in from hospital and have distinct needs, with notable dependency and frailty (Burton et al. 2022).

Considering the limited evidence to inform practice, this team initially performed a case note review of people admitted to hospital from home and discharged to care home. That research identified significant variation in the care, treatment and experiences of this population (Harrison et al. 2017; Rhynas et al. 2018). This identified complex decision‐making that was time‐consuming, involved varying stakeholder perspectives and limited documentation of the person's voice. The aim of this follow‐up study is to explore how decisions are made to discharge older people directly from acute hospital to care home. It considers the perspectives of the person and other relevant stakeholders in the decision‐making process, exploring the factors influencing decisions and how they are communicated.

## 2 Research Design and Methods

### 2.1 Research Design

**FIGURE 1:**
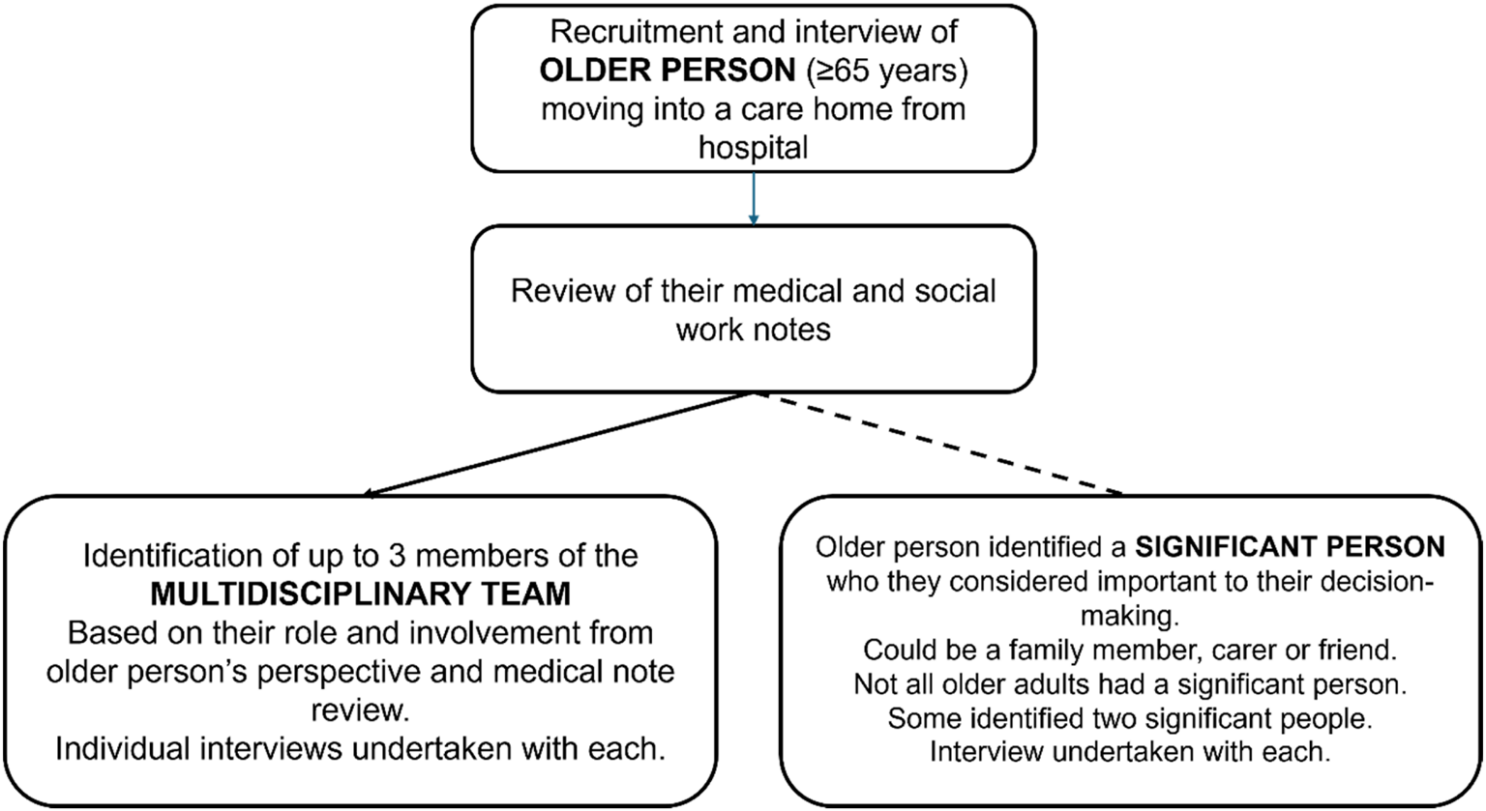
Composition of individual datasets.

A case study design was used to enable exploration of stakeholders' contributions to the decision‐making process. Case study research uses a range of data sources to explore phenomena from different perspectives (Keen and Packwood 1995). This permits insight into the explanatory processes and understandings that shape the complex interactions in care home discharge decisions (Crowe et al. 2011). The approach taken was to have a single case study with embedded units (Baxter and Jack 2008). Construction of the case study ‘discharge to care home from the acute hospital’ uses data collected from individual datasets, comprising the following (Figure 1):

### 2.2 Methods

Ethical approval was received from the NHS Research Ethics Committee. The study team was a nurse (GS) (BSc(Hons), MN), a trainee in geriatric medicine (JKB) (BSc(Hons), MRes, MBChB) and a nurse academic (SJR) (BSc(Hons), MSc, PhD). All were experienced in communicating with older people in a clinical and academic capacity but with no care responsibilities in the study setting or prior relationship with participants. Two researchers (GS, JKB) conducted recruitment at two acute hospitals in Scotland, in the Acute Older People and Stroke departments (June 2017–May 2018). The Acute Older People wards (*n* = 5) offer acute and/or complex medical assessment and intervention and/or multidisciplinary rehabilitation to over 65 s. The Stroke ward (*n* = 1) offers people of any age requiring acute management and multidisciplinary rehabilitation for stroke.

Rapport was built through ward visits and initial approach to potential participants included informal conversation, information giving and opportunity to ask questions. Information sheets were left with the person, and the researcher returned to take written informed consent within 24–48 h of information giving. A cooling‐off period of 24 h was left before the interview. Assent was reconfirmed at the time of the interview. Older people were invited to have someone significant to them present at any stage, including the initial introduction of the study.

The datasets comprise hospitalised older people admitted from home for whom the decision had been made during the current admission to move directly into a care home. A purposive sample is included representing variation (Bryman 2016) and a range of characteristics arising from the team's previous case‐note review (Harrison et al. 2017; Rhynas et al. 2018) including family involvement, dependency, and level of pre‐admission support. Researchers invited older people face‐to‐face to participate after they had made the decision to move to a care home, and interviews took place in the period between decision‐making and hospital discharge. Only older people that the MDT considered to have capacity for discharge decision‐making were approached for inclusion. Capacity to consent was formally assessed by the researchers based on the definitions provided in the Adults with Incapacity (Scotland) Act (2000). The older person decided if they had significant person(s) and identified which MDT members had been involved in decision‐making and should be approached. MDT members highlighted by the older person were identified through health records.

A semi‐structured interview guide was developed, piloted and refined and used for all interviews (Appendix S1). Two female researchers (GS, JKB) undertook interviews, with the same researcher conducting interviews for a single dataset. Interviews were carried out in English and in the hospital setting or for some significant persons, their homes. Significant people and MDT members were interviewed individually during the older person's admission. Only researchers and participants were present during interviews. Interviews, ranging from 30 to 60 min, were audio‐recorded and transcribed verbatim by NHS transcription services and checked by the study principal investigator (GS). Researchers made notes during interviews. No repeat interviews were undertaken. The data were securely stored in line with NHS data management practices.

Inductive thematic analysis (Braun and Clarke 2006) within the context of case study design was performed in NVIVO Version 11. To create an overall single case, within case and cross‐case analyses was performed (Baxter and Jack 2008). The first three stages of Braun and Clarke's (2006) approach were used within each dataset, with stages four and five completed across the datasets. Two researchers (GS, JKB) coded the transcripts independently within each dataset and then codes, themes and definitions were discussed, reviewed and confirmed among the study team. Codes and themes were refined and individually applied to each dataset followed by the data as a whole. Within case analysis ensured perspectives from each stakeholder were fully explored. Cross‐case analysis ensured similarities and differences between the datasets were made explicit and themes common to all datasets could emerge.

### 2.3 Quality and Rigour

The study has been reported in accordance with accepted practice for qualitative research (Tong et al. 2007) (Appendix S2). Rigour in qualitative research relies on the four criteria of credibility, dependability, confirmability and transferability (Lincoln and Guba 1985). These strategies can be specifically applied to case study research (Houghton et al. 2013) to ensure the trustworthiness of the findings. It is recognised that personal interpretations may differ; therefore, emphasising the importance of presenting transparent decision‐making processes to enhance trustworthiness (Johnson et al. 2020; Nowell et al. 2017), outlined in Table 1.

**TABLE 1:** Rigour in this case study research.

Approaches to rigour	Strategy
Credibility	One researcher worked with the participants from each dataset to ensure prolonged and extended engagement Data triangulation was achieved by gaining three different perspectives on each experience—older person, family member and professional Analytic triangulation: Each dataset was analysed independently by two researchers before discussion with the wider study team. Consensus was reached within the team when differences in interpretation occurred. This process of peer debriefing contributed to analytic rigour and allowed the team to have confidence in the findings No participant member checking was undertaken. It was judged as burdensome for the older people participants to share transcripts at the time of moving home and we do not wish to introduce bias by preferentially sharing transcripts with other stakeholders and not older people, in case this prioritises other voices
Dependability and confirmability	Each dataset was analysed independently by two researchers and the resulting codes were discussed and fully described in a code book to reflect shared understandings The research team reflected together on the data throughout analysis and on the extracts chosen for presentation Audit trail of the research process recorded in NVivo and subsequently in reporting
Transferability	The data extracts chosen for presentation at conferences and within this publication allow readers to hear the words of participants in some context which permits judgements to be made about researcher interpretations (Johnson et al. 2020)

### 2.4 Participant Characteristics

**FIGURE 2:**
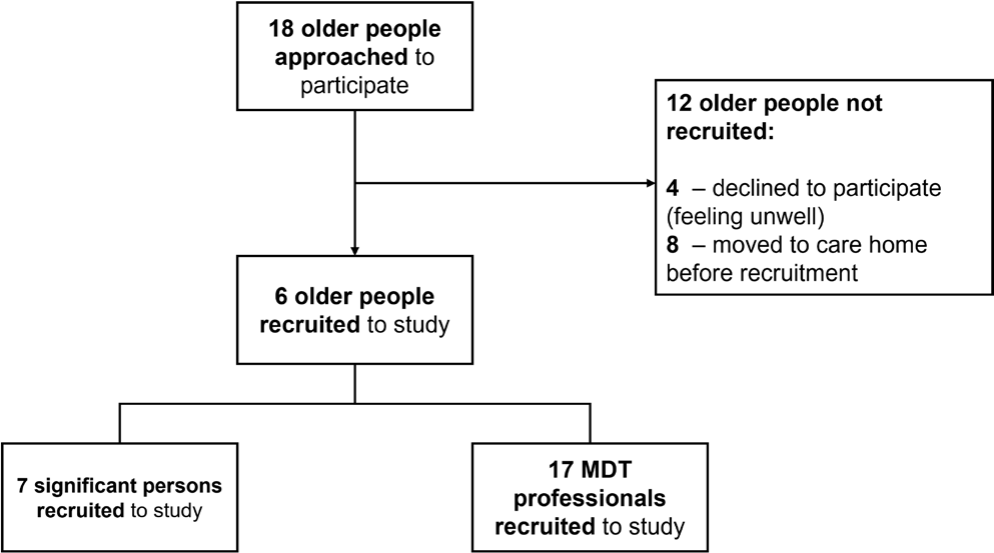
Overview of recruitment.

Interviews were conducted with 30 participants, forming six complete datasets (Figure 2). As a small‐scale project, data collection ended when sufficient data had been collected to address the research questions within the timeframe of funding.

Table 2 summarises key characteristics for each dataset. The significant people recruited to this study were family members and therefore the term ‘family member’ is used throughout the results. Pseudonyms have been ascribed to the older person, with the remaining participants described by their relationship to that person or their professional role.

**TABLE 2:** Characteristics of study participants.

Pseudonyms	Characteristics of patient participant and reason for admission	Other dataset participants	Key factors in decision‐making
Agnes	89‐year‐old female History of dementia Admitted with acute stroke	No next of kin Occupational therapist Consultant Social worker	Social isolation Being ‘looked after’
Harry	78‐year‐old male History of strokes Admitted with acute abdomen	Nephew Physiotherapist Consultant Social worker	Preserving quality of life Availability of family support Long hospitalisation Prior disability uncovered
Isa	94‐year‐old female History of progressive sight loss and multiple hospitalisations Admitted after injury to leg and cellulitis	Daughter and granddaughter Junior doctor Staff nurse Social worker	Loss of sight Availability of family support Recurrent admissions
Peter	94‐year‐old male History of heart failure Admitted following falls	Daughter Consultant Charge nurse	Safety Reducing family distress Long hospitalisation
Arthur	70‐year‐old male Had become housebound over prior months due to worsening mobility Admitted following falls	Sister Consultant Physiotherapist x 2	Social isolation Availability of family support Acceptance of professional help Safety
Robert	67‐year old male Previously independent Admitted with acute stroke	Partner and her son Consultant Advanced nurse practitioner Social worker	Acute change Availability of family support Long hospitalisation

## 3 Results

Three core themes were developed from the analysis, namely rationalising the decision, the context of the decision and complexities in communication. We found that the rationale for the discharge decision was debated within the context of participants' relationships, with expectations of support, perceptions of risk and future aspirations shaping the rationale presented. The hospital provided a specific context which both facilitated and inhibited decision‐making. There were complexities in communication both with the older person and MDT, where professional boundaries were constraining, and older people's desires to talk about their futures were limited.

### 3.1 Rationalising the Decision

The rationale for the decision to move into care home is explored and debated within the context of participants' relationships. This encapsulates deciphering responsibilities for the older person's support needs, debating perceived and actual risks of returning home and considering the benefits of moving into care home. Findings demonstrate the relational complexities that influence both the making and framing of decisions.

#### 3.1.1 Personal, Family and Professional Expectations of Support at Home

In datasets where the family member is the person's own adult child, there is, at times, a complex, sometimes tacit expectation from the person that the adult child will provide support should they return home. In some instances, it is realised that this expectation would be unmet, requiring re‐adjustment and reframing of expectations.

Isa: ‘My age, hen, and giving my daughter some life now…. Well, let her live her own life with her grandson. And I just seem to be in the road’.

The data demonstrate strong expectations from the MDT that the role of the family includes supporting the person at home. While this expectation is often implicit, it plays a central role in decision‐making. Where families are not able to provide or increase support, care home becomes the recommendation from professionals.

Agnes’ Consultant: ‘I think it would have needed probably an increase in her already substantial package of care… I think with the support of her family that would have been feasible’.

Many families provided support to the person prior to admission. This is not reported as intolerable, and none demonstrate resentment despite some highlighting the associated challenges. However, where the person requires increased family support, concerns relating to this increased commitment emerge. Findings highlight an implicit judgement by the MDT when this increased care cannot be supported.

Arthur’s Physiotherapist: ‘If the circumstances would have been different and his sister would have been heavily involved and readily involved, then no, we could have supported him at home. But he just lacked the support….’

The MDT make their views explicit when they perceive families to be taking on support that is too burdensome.

Robert’s Partner: ‘And the consultant said to me…“I notice”, he says, “that you're going to take him home to care for him”. I went, “aye”. He says, “it's not an option”. He says, “he needs far, far too much care”’.

In instances where the MDT recommends a care home, the influence that the interaction between the MDT and family can have in decision‐making becomes evident.

#### 3.1.2 Mitigating the Risks of Returning Home

In several of the datasets, findings suggest the person makes the decision because they feel it would lessen the family burden.

Arthur: ‘Just to be safe, be safe and no be a trouble to my family…’

However, in some instances the person recognises that moving into a care home could also prevent unwanted future hospital admissions.

Peter: ‘Mainly because the family were worried about me, and I didn't want that. I didn't like the idea of going into a care home, but I realised it probably was the only way to stay out of hospital…’

The person acknowledges they can no longer manage at home and the expectation develops that a care home could alleviate this.

Harry: ‘…overall it was always coming to the fact that I couldnae live by myself, back home, so it hit me it was the main option, which I agree’

Findings suggest older people can be presented with a proposal that lacks feasible alternatives to care home, and this presents the person with limited choice over a decision that they are then encouraged to make and own themselves.

Isa’s Daughter: ‘…basically your only option…for her to stay at home, she's talking about having someone there practically all the time to make sure she's alright. And they don't do that…it's not possible.’

Safety and risk prevention add complexity to the relationship between the adult child and parent. The relative's rationale often centres around the risk of harm and a focus on the worst‐case scenario (e.g., falling and lying on the floor for many hours). While in some instances the existence of risk is real, the focus overwhelms any consideration of the many potentially uneventful days. The family member rationalises the decision by framing it in relation to risk, with risk aversion often outweighing the risk itself.

Isa’s Daughter: ‘She stays in [place] which is an hour and a half from us…Which is fine…we've been doing it for years…But if in an emergency, it's not really been advisable. I can't just say, “Right, Mum, I'll be there in five minutes”’.

Peter’s Daughter: ‘We just felt he wouldn't manage at home and we would just be worrying about him all the time.’

Family members consider moving to a care home as mitigating the risks of returning home. The concept of risk is absent from their discussions focused on moving to a care home, implying they see little risk associated with care home living. In contrast, many of the professionals acknowledge that the risks (e.g., falls) remain in the care home setting.

Arthur’s Physiotherapist: *‘*…everybody falls. They can fall in a nursing home….They might as well be at home…. Nobody is supervised 24/7. So, we would never, ever make this a main criteria….it's a normal and honest chat with the family to say, “yes your mother will fall most probably. But this will happen in any environment. So it's your decision essentially but we can tell you now that…nursing home admission is not a falls prevention”’.

For the family member, the overall responsibility for the person's safety and well‐being appears to be relinquished through the move into a care home, and with this comes a relief from the associated worry. However, the professionals do not appear to appreciate the psychological burden of responsibility that relatives feel, potentially demonstrating an imbalance between professional and family perceptions of risk.

#### 3.1.3 Positive Aspirations for the Future

None of the family members highlight concern about the person's well‐being in the care home. Instead, moving to a care home is associated with positive aspirations and offering potential for the person to thrive.

Arthur’s Sister: ‘We could go and get him and take him to the pub…he'd still be able to go on holidays…take him round to his house…. sit him in the garden, do all the sort of stuff that he used to do, but with help’.

Many family members acknowledge previously holding negative opinions of care homes; however, their attitudes shift towards the belief they offer a solution for unresolvable issues in the community. For some, returning home is associated with restrictions such as the inability to go out alone, difficulties managing continence or immobility between care visits. Care homes offer a degree of freedom through the provision of round‐the‐clock care, social stimulation and opportunity to be around like‐minded people. For some family members, a care home will permit opportunities to spend quality time with the person.

Peter’s Daughter: ‘We want somewhere local so if I'm just out shopping, I can think “…I can just pop in and see him”, you know so we can dip‐in‐and‐out as often as we want’.

These family members remain committed to maintaining and potentially extending regular contact beyond the move, allowing professionals to deliver the additional help needed.

Some individuals make the decision to move to a care home with the understanding that it is a temporary arrangement.

Robert: ‘Well, what I'm told happens, is I need to go to a care home.…or some sort of nursing home. But that is just like a sort of holding pen, until they think I'm able to go home, until they think I'll manage at home, and all the care package is in place’.

Robert’s Social Worker: ‘when I spoke to him….I was thinking ‘he thinks it's only going to be for a short time’. And again, the way I would play it is that we do our review after 12 weeks. So, when I go back, if he expects to go back home, then we have that conversation at that point’.

For some older people in this study, it is unlikely that the decision to move into a care home could be reversed in future as their care needs are unable to be supported in the community.

### 3.2 The Context of the Decision

The second theme emphasises the significance of the hospital context for decision‐making through its opportunity to offer time, support and access to professionals to explore and plan the future; yet this context can inhibit preferences during decision‐making.

#### 3.2.1 Time and Distance

For many, the admission provides time and distance for the person and their family to consider the reality of their circumstances. For some, this highlights just how challenging life had become at home. For others, the decline the person experiences during their admission means their needs are too great to return home, initiating conversations about care home. Being in hospital facilitates these conversations, with help from professionals to elicit concerns and consider alternatives.

Peter’s Daughter: ‘So I would say to the girls [nurses], you know, “how has he been, how much are you having to do?…what are your thoughts? Do you think he would manage at home?” So, I did ask the question quite often with various members of the team who all felt he would struggle at home’.

The time–pressure often associated with making this decision in hospital is not apparent in this study. Professionals acknowledge the pressures associated with decision‐making timeframes, and the need to discharge people when they are medically well. However, neither interviews nor case notes report time pressure overtly influencing the older person or family members in their decision‐making. Findings reveal evidence of measures to avoid the constraints of the system, such as professionals delaying formally documenting somebody as ‘medically ready for discharge’ or stating the older person should not be boarded to other wards.

Peter’s Nurse: ‘…Although he was medically precarious, he was ready for discharge. We just said, “not ready for discharge, discharge destination not decided”….Then when the decision was [formally] made …, because of the emotional sort of journey he went through, … he was not suitable to board’.

These measures provide additional time for individuals to reach and process their decision.

#### 3.2.2 Inhibiting Preferences

Findings suggest the context can limit specific care home choice and potentially prevent preferences being realised. Many of the participants invest time and emotion choosing a care home. For both Agnes and Harry, decision‐making is framed in terms of one specific care home, with neither conceptualising their decision more widely.

Agnes: ‘I told him I would like to go to [care home name]. Nowhere else but [care home name], because it's near hand and everything…’

Harry’s Social Worker: *‘*Unfortunately, places like [Harry's care home choice] have huge waiting lists, so he wouldn't have been allowed to stay in hospital to await the bed at [care home], because it could be months, years, that a bed at [care home] would become available…’

Due to the popularity of the chosen care homes, both Agnes and Harry are required to move into interim care homes to either await their choice of home or choose from alternatives.

Agnes’ Social Worker: ‘So again we generally ask people to make three choices. We explain the moving on policy. Because obviously once somebody's medically fit, we're trying to get them out of hospital as quick as possible. So, it may be the person is offered an interim placement until their choice of care home becomes available’.

At times, there is a lack of transparency from professionals around the likelihood of the chosen care home meeting discharge timescales and care home assessment criteria when initial choices are being made.

### 3.3 Complexities in Communication

The final theme offers insight into the complexity of communication surrounding decision‐making about moving into a care home from hospital.

Professional boundaries, uncertainty and lack of role clarity impact what is communicated with the person and their family. Many older people want the opportunity to talk through their decision, develop a new narrative, share their feelings and seek reassurance. From the perspectives of the professionals interviewed, these conversations regularly occurred, and the records reviewed support this. However, some older people report not remembering conversations having taken place.

Robert’s Social Worker: ‘He [Robert] was quite keen to go home. And that's maybe because he didn't have that discussion with the ward staff. It's documented as being a discussion….quite a fleeting discussion….you know, when the consultant comes to the bedside. And they'll talk in, kind of, general terms about how they are and then they'll say something like…“do you think you could manage at home?” And depending on what they say at that moment, that will be counted as the discussion…for a lot of people that's just chitchat with the doctor. It's not really a discussion about the future’.

Many professionals objectively recognise the magnitude of the decision but do not engage with families in a manner that acknowledges this enormity. In some instances, the professionals avoid bringing the topic up in case it causes the person to become distressed or change their mind. Others report role uncertainty impeding communication and indicate they often defer to professionals whom they perceive as having greater expertise. Interprofessional roles and responsibilities around care home are found to be unclear, which impedes collaboration between those involved.

Harry’s Consultant: ‘I'm sometimes involved in the initial discussions with social work and family…I don't feel confident enough, I don't know the details and I don't think it's…necessarily my role to delve into those details with them’.

The level of discussion about the decision between the family member and the MDT is also reported to be limited.

Harry’s Nephew: ‘I don't think it's been particularly spoken through with [Harry] particularly well, certainly not with me. I don't think either of us know the next steps, what the route is, what the consequences of that are, the financial implications, never mind the medical or his wellbeing. I don't know any of that and neither does [family member], so I would have to say shambles is how it is being handled’.

Many of the family members feel relief that the decision has been made, uncertainty about the future, and guilt about whether it is the right decision. However, there is little opportunity to communicate these feelings with the team.

Harry’s Nephew: ‘…what could have made it helpful, better? Simply brief discussions like this, a brief meeting so that [Harry] was very clear about what the next steps were’.

The healthcare records offer an overview of the decision‐making process yet often lack detail of the iterative nature of the discussions, omitting evidence of ongoing conversations after the decision has been made.

Peter’s Nurse: ‘People might say, “updated daughter”, but they won't have gone into the whole ream of…the doctor's probably said “discussed options of care home” but they wouldn't have gone into…and part of that is time… some of these conversations are sort of in an informal way, they're sort of as you're helping him walk to the toilet, “how are you feeling today?” And people probably don't see it as an event to document which probably is the wrong thing, but that's the reality’.

While there is evidence of these conversations in social work records, these are not easily accessible to hospital‐based practitioners. Some healthcare professionals suggest that only care needs, concerns and issues relating to risk, uncertainty or inconsistency surrounding the decision should be documented.

## 4 Discussion

This study commenced from the widely recognised United Kingdom (UK) policy perspective that moving to a care home from an acute hospital should be avoided (Scotland 2014; Scottish Government 2023), and people should not have to make life‐changing decisions in a time of crisis (National Institute for Health and Care Excellence 2015). Research undertaken internationally supports the alternative view that the crisis itself often catalyses these difficult decisions, prompting a loss in the person's confidence to remain at home and families questioning their capacity to support the person's needs (Fekonja et al. 2021; Lam et al. 2025). Findings from this study indicate the struggles in caring relationships and highlight the hospital context in providing access to professionals and structures to facilitate consideration of care home as a destination and potential resolution of the challenges experienced. The impact of individual, familial and system pressures, in addition to varied perceptions and experiences on decision‐making processes, were evident in this study. Findings emphasise concepts of risk/benefit relating to care home transitions, as well as how communication is integral to the experiences of those involved. The complex and personal nature of decision‐making about long‐term care in hospital was indicated in previous work (Harrison et al. 2017; Rhynas et al. 2018). This study captures the perspectives of different stakeholders, highlighting the need to prepare them for the significance and complexity of the situation.

The idea of care home transition was an uncomfortable discussion point for participants, evidenced by professionals' discomfort in having clear and signposted discussions. Findings demonstrate limited perceived opportunities for discussing options, preferences and viable alternatives. In a study exploring older people's experience of transition to and life within care home, findings suggest limited involvement in discussions about the decision can leave older people feeling disempowered (Pocock et al. 2020). During a period in which a person may experience minimal control over their deteriorating health, Zhang et al. (2025) note the importance of the older person's involvement in care transition decision‐making to promote a sense of autonomy. Our findings indicate uncertainty among professionals about what to communicate, coupled with lack of role clarity, significantly reduces professionals' readiness to communicate with families. Professionals' reluctance to initiate or revisit discussions about decision‐making, fearing this would cause additional distress, appeared to negatively impact decision‐making experiences. Evidence of uncertainty regarding discharge planning processes, regardless of destination, aligns with existing literature noting a detrimental impact on professional communication and discharge coordination (Redwood et al. 2020; Zhang et al. 2025). Lack of role clarity is an identified barrier to effective discharge planning, leading to miscommunication and tensions in inter‐professional relationships (Nosbusch et al. 2010). Challenges to autonomy and agency are more evident in hospital; with concerns over health, stress and unclear communication compounding challenges to the overall decision‐making process (Lilleheie et al. 2019).

The range of emotions experienced by the person and their family is evident in this study, supporting existing research undertaken in the UK and internationally emphasising the potential for guilt and fear (Fekonja et al. 2021; Robinson and Fisher 2023; Zhang et al. 2025), along with emotional distress (Argyle et al. 2010) to be experienced during the decision‐making process. Findings in our study emphasise that these emotions often appear to go largely unrecognised by staff. The role that risk can play in initiating decision‐making about care home admission is reported elsewhere (Cole et al. 2021). Our findings contribute to this, demonstrating the psychological burden families feel when considering risk. Fekonja et al. (2021) emphasise that the emotional turmoil often experienced by families during decision‐making is only relinquished once their loved one has moved into a care home and they observe that their needs are now being met. By contrast, Robinson and Fisher (2023) suggest that the guilt and sadness experienced by family members can continue in the months and years following the move; although juxtaposed with a sense of relief due to the person now being safe and looked after (Robinson and Fisher 2023). Findings in our study add to this, demonstrating that families hold aspirations for this relief and associate the older person's move into a care home with potential freedom from the responsibility for the person's well‐being. From the perspective of the older person themselves, Samsi et al. (2019) suggest that moving into a care home can be associated with a reduction in their level of independence, perceived as being a more restrictive environment. By contrast, our findings indicate the potential for older people to anticipate that a care home could offer freedom and independence, particularly from the struggle of self‐care and feeling a burden to their family.

The hospital environment allowed families in our study to broach and discuss the idea of moving to a care home. However, lack of familiarity with the care system, lack of capacity in a chosen care home and lack of communication with staff impeded the process itself. The Scottish Government (2023) outlines that while the person's choice of care home should be facilitated, accommodation availability timeframes may limit this aspiration. Samsi et al. (2022) note that awareness of vacancies in a preferred care home can be a catalyst for decision‐making. Further, Cole et al. (2018) emphasise that choosing the right care home is a significant component of the decision, with limited guidance when choosing a care home or limited choice of care home associated by carers with a lack of support during the process. In our study, the availability of chosen care homes was more limited than portrayed by professionals and the impact on those involved is evident, making the process complex, emotional and lengthy.

## 5 Strengths and Limitations

This study successfully recruited participants in the acute hospital to share perspectives on this under‐researched topic. Previous studies focused on seeking the views of those not immediately facing the decision (Dubois et al. 2008). By exploring decision‐making with different stakeholders, this study offered the opportunity to contrast professional, individual and family perspectives of the decision‐making process, highlighting the challenge of balancing different stakeholder views and prioritisation of the person's well‐being.

A notable limitation in the methods of recruitment is the potential for selection bias. Interviews were not undertaken with all those involved in each older person's care, and the older people prepared to participate may have different perspectives from those who declined or who could not be included. In addition, participants could only be included in the study if they were assessed as having the capacity to consent to participate, as stipulated by the approving ethics committee. This is an all‐too‐common issue (Shepherd et al. 2019), whereby permission is not granted by research ethics committees to include those with altered capacity. The majority of those in hospital awaiting care home admission have a cognitive impairment (Burton et al. 2018) and many are not considered to have the capacity to make discharge decisions. The insights from those who have the capacity may not be applicable to a more vulnerable population, who are often denied opportunities to contribute to discharge decision‐making (Kelley et al. 2021). This study sought to be inclusive of any person that the older people considered significant to their decision‐making, which may have included a family member, carer or friend. However, all significant persons recruited to this study were family members, which limits understanding of the views, perspectives and involvement in decision‐making of people that hold a different type of relationship with the older person.

Due to study time‐frame, data saturation has not been claimed. However, through employing qualitative approaches including participant interaction and deep immersion in the data, it was possible to identify insights from the data generated that offer plausible explanations about how decisions to move into a care home from hospital are made (Johnson et al. 2020). An additional limitation arises from the absence of member checking. It was deemed inappropriate to approach older people for this purpose beyond their move into care home; and completing member checking with a partial sample risks introducing bias by preferentially involving only those stakeholders' voices available for this (Lloyd et al. 2024).

This study was undertaken in 2017–2018, with the evident delay in publication offering a further limitation. However, discussion of the findings with reference to more recent research undertaken in the UK and internationally emphasises that the complexities inherent in decision‐making about moving into a care home remain prevalent today. Since this study was undertaken, there remains a lack of research focused on moving into a care home directly from hospital, despite this continuing to be a location from which this transition occurs (Harrison et al. 2017; Rhynas et al. 2018; Scottish Government 2023). This study offers useful insight into how this complex decision is experienced by older people, their families and health and social care professionals in this context.

## 6 Implications for Practice, Education and Research

Our study has significant implications for practice, education and research for decision‐making about moving into a care home from hospital.

There is a clear need for interdisciplinary education about care home discharge, and the importance of professionals' availability and approachability throughout decision‐making. It is essential that the MDT recognise the different roles involved in decision‐making and acknowledge a shared responsibility for the clear provision of information and guidance (Scottish Government 2023). Zhang et al. (2024) emphasise the importance of both health and social care professionals having a comprehensive understanding of care transition processes to facilitate effective and timely information provision and support. Connolly et al. (2009) suggest enhanced understanding of inter‐professional roles may reduce tensions in MDTs surrounding discharge planning and encourage a collaborative personalised approach. Improving role clarity may also enhance communication with those involved, enhancing the overall experience of decision‐making (Connolly et al. 2009). However, there remains minimal guidance regarding how to engage older people in decision‐making about long‐term care, especially in the hospital setting (Durocher et al. 2019), emphasising a clear area for future development.

Professionals should recognise and be sensitive to the significance of this oftentimes final decision, including the emotional impact of the decision on those involved (Zhang et al. 2024). There is a need for greater advocacy for older people making this decision, creating opportunity for their views to be expressed, enhancing their sense of control (Pocock et al. 2020). In addition, findings emphasise the importance of professionals identifying how risk can be discussed and managed in accordance with the person's preferences (Atwal et al. 2012). Within the constraints of a pressurised health and social care system, time should be afforded to older people for reflection and discussions which are subsequently documented within the health and social care records (Ellis and Sevdalis 2019).

This study has emphasised the significance of the availability and choice of care home to the overall decision‐making process. Availability should not be misrepresented by professionals, preventing unrealistic and unmet expectations and ensuring decisions are not made contingent on specific care home availability. Professionals have a responsibility to be transparent about the availability of specific care homes and the possibility of interim transfer to alternative facilities to await the chosen care home (Scottish Government 2023).

Future research must involve those with altered capacity and their families to understand experiences and ensure care pathways support decision‐making. A common theme during recruitment was the identification of older people who were going home, but for whom the MDT thought care home admission was a likely future outcome. This poorly understood group should be a focus for future research around supporting moves without requiring further hospitalisation. There remains a UK policy and practice disconnect around the frequency of moves into care home from hospitals. There is a need to ensure policy recommendations are updated to reflect experiences from research and practice, to ensure older people do not face unnecessary barriers to accessing the care and support they need. There is also limited understanding of the transition from the perspective of those older people newly arrived in a care home; therefore, research retrospectively exploring the person's experiences could offer an opportunity to enhance preparation during hospital admission.

## 7 Conclusion

This study explored experiences of decision‐making about discharge to care home. Findings uncover a complex process requiring interdisciplinary communication and role clarification. Older people making this decision would like greater interaction from professionals to discuss and explore their feelings. The significance of the decision for older people and their families is currently under‐recognised by professionals. The need for care home admission continues to grow (Kingston et al. 2017), thus the importance of improving practice in this area cannot be underestimated.

## Author Contributions

**Gemma Stevenson:** overall coordination of the study (principal investigator), conceived the study design, collected the data, analysed the data and wrote the manuscript. **Jennifer Kirsty Burton:** conceived the study design, collected the data, analysed the data and wrote the manuscript. **Susan D. Shenkin:** methodological and intellectual review and editing of study design, project management guidance and contributed to manuscript editing. **Juliet MacArthur:** methodological and intellectual review and editing of study design, management support and supervision during the research and contributed to manuscript editing. **Brendan McCormack:** methodological and intellectual review and editing of study design, project management guidance and contributed to manuscript editing. **Clare Halpenny:** involved in the writing of the manuscript. **Sarah Rhynas:** conceived the study design, provided management support and supervision during the research process, oversaw data collection, was involved in data analysis and wrote the manuscript.

## Ethics Statement

Ethical approval was provided by the West of Scotland 4 Research Ethics Committee on 26 April 2017 [REC 17/WS/0067]. NHS Research & Development approval was granted on 22 May 2017 from NHS Lothian [2017/0123]. All participants provided written, informed consent prior to undertaking data collection. Ongoing consent was obtained prior to the start of semi‐structured interviews. Participants were informed that their participation was voluntary, and they had the right to withdraw their consent at any stage and it would not affect the care or treatment they received.

## Conflicts of Interest

The authors declare no conflicts of interest.

## Supporting information


Appendix S1.

Appendix S2.

## Data Availability

The data that support the findings of this study are available on request from the corresponding author. The data are not publicly available due to privacy or ethical restrictions.
