# Supplementary material for: One Chance to Get it Right: Exploring Perspectives and Experiences in Care Home Discharge Decision‐Making in the Acute Hospital
Source: Int J Older People Nurs. 2025 Sep 5;20(5):e70041. doi: 10.1111/opn.70041 (PMC12412644; doi:10.1111/opn.70041)
Supplement: Supplementary file 1 — Appendix S1. [file OPN-20-e70041-s002.docx]

**APPENDIX 1: INTERVIEW GUIDE**

**Older Person**

**Pre-admission circumstances**

1) Can you tell me about your home circumstances – who was at home with you?

2) And do you have any (other) family members or friends nearby, or that are in regular contact with you?

3) Can you tell me about any additional support that you had prior to your hospital admission?

**Admission circumstances**

1) What happened that meant you had to come into hospital?

2) Can you tell me more about how you felt when you first came into hospital?

3) Have you had any family or friends coming in to visit you?

**Discharge circumstances**

1) Can you tell me what is happening when you leave hospital?

2) How do you feel about this?

3) During your time in hospital, have your family or friends, or someone else significant to you spoken to you about what will be happening when you leave hospital?

4) During your time in hospital, have any of the staff asked spoken to you about what will be happening when you leave hospital?

5) Do you feel that you got enough opportunity to talk about your future plans or to weigh up the best options?

6) When thinking about your future plans, what is your main priority?

7) Did you agree with the things that the staff or your family said about you and your future plans?

8) What do you think was the main reason for this decision being made?

9) How do you feel about how the decision was made?

10) Did you feel part of the decision?

11) Who did you find the most helpful during the decision-making period?

12) Is there anything that would have made the decision-making easier?

13) Did you (or are you going to) get an opportunity to visit your previous home during your hospital stay?

14) Have you visited the care home yet? Or has someone you trust been to visit for you?

**Significant Person**

**Pre-admission circumstances**

1) Can you tell me about (older person’s name)’s home circumstances – who was at home with them?

2) And do they have any (other) family members or friends nearby, or that are in regular contact with them (including yourself)?

3) To your knowledge, did (older person’s name) have any healthcare conditions that was causing them difficulties at home?

4) (If details provided in question 3) How do you feel they were able to manage with these difficulties?

**Admission circumstances**

1) What happened that meant (older person’s name) had to come into hospital?

2) Can you tell me more about how you felt about (older person’s name) coming into hospital?

3) How have things been during their time in hospital?

**Discharge circumstances**

1) Why do you think the decision was made for (older person’s name) to be discharged to a care home?

2) How do you feel about this?

3) Was a care home always something you considered?

4) What was the main reason for this decision being made?

5) What was your main priority when making this decision?

6) What is your understanding of what (older person’s name) wanted?

7) Can you tell me more about how this decision was made?

8) To your knowledge, who was involved in this decision?

9) What factors do you think influenced who was involved in this decision?

10) How do you feel about how the decision was made?

11) How have you been involved in this decision?

12) Who supported you most during this period of decision-making?

13) What could have made the process easier for you?

14) What were your priorities when you were contributing to this decision?

15) How did you balance your own feelings with (older person’s name) feelings about a care home?

16) How do you foresee this decision changing life for you and (older person’s name)?

17) What were your priorities when choosing the specific care home?

**Multidisciplinary Team Member**

**Pre-admission circumstances**

1) Can you tell me what you know about their social circumstances prior to admission – who was at home with them, and what kind of social support they received (be it from family, friends or other)?

2) To your knowledge, did (older person’s name) have any healthcare conditions that were causing them difficulties at home?

**Admission circumstances**

1) Can you tell me about why (older person’s name) were admitted to hospital?

2) During (older person’s name) time in hospital, can you tell me about your involvement with them – what role have you played in their care?

3) And how have (older person’s name) been during their admission?

**Discharge circumstances**

1) Why do you think the decision was made for (older person’s name) to be discharged to a care home?

2) What is your understanding of what (older person’s name) wanted?

3) Can you tell me more about how this decision was made?

4) What factors do you think influenced how the decision was made?

5) What role did you play in this process?

6) What was your main priority when making this decision?

7) Who else was involved in this process?

8) What factors do you think influenced who was involved?

9) What things did you specifically consider in making your recommendations for (older person’s name)?

10) Did you feel that all parties were in agreement with this decision?

11) How did you balance the wishes of the family and older person with your own expertise during this decision-making process? Is this challenging?

12) What was your main reason for recommending discharge to a care home?

13) At what point of the admission did you first consider care home for this older person?

14) What has been done to help prepare (older person’s name) for the move to a care home? Do you think that preparation for this is part of your role?

15) Is there anything that could have been done differently?
