# Supplementary material for: One Chance to Get it Right: Exploring Perspectives and Experiences in Care Home Discharge Decision‐Making in the Acute Hospital
Source: Int J Older People Nurs. 2025 Sep 5;20(5):e70041. doi: 10.1111/opn.70041 (PMC12412644; doi:10.1111/opn.70041)
Supplement: Supplementary file 2 — Appendix S2. [file OPN-20-e70041-s001.docx]

**APPENDIX 2: COREQ Checklist**

**The Consolidated Criteria for Reporting Qualitative Studies (COREQ) – 32 item checklist** (Tong et al., 2007)

| **No Item** | **Guide Questions/Description** | **Response** |
| --- | --- | --- |
| **Domain 1: Research Team and Reflexivity** | | |
| Personal Characteristics | | |
| The study team comprised of a nurse (GS), a trainee in geriatric medicine (JKB) and a nurse academic (SJR) with experience in working with older people in a research and clinical capacity. | | |
| 1. Interviewer | Which author(s) conducted the interviews? | All interviews were conducted by (GS) and (JKB).  All interviews within each dataset were conducted by the same author. |
| 1. Credentials | What were the author’s credentials? | JKB: BSc(Hons), MRes, MBChB  GS: BSc(Hons), MN  SJR: BSc(Hons), MSc, PhD |
| 1. Occupation | What was their occupation at the time of the study? | JKB: Trainee in geriatric medicine, pre-doctoral fellow.  GS: Registered Nurse and Senior Research Nurse.  SJR: Registered Nurse, Lecturer |
| 1. Gender | What was their gender at the time of the study? | All members of the study team were female. |
| 1. Experience and training | What experience or training did the researcher(s) have? | JKB: had experiencing communicating with people receiving care, families and healthcare professionals in their clinical training (12 years). In addition, JKB had Masters-level training in qualitative research.  GS: had experience in both quantitative and qualitative research methodologies and methods, and research study coordination through her role as a Senior Research Nurse.  SJR had experience with qualitative research methodologies and methods. In addition, SJR had experience as a Principal Investigator and PhD supervisor. |
| Relationship with Participants | | |
| 1. Relationship established | Was a relationship established prior to study commencement? | Rapport was built through ward visits. Initial approach included informal conversation, information giving and opportunity to ask questions. Information sheets were left with the person at this stage. Researcher returned to take consent within 24-48h of information giving. Cooling off period was then given with 24h left before interview. Assent was reconfirmed at time of interview. Older people were invited to have someone significant to them present at any stage, including the initial introduction of the study.  The research team had no care responsibilities in the setting or prior relationship with the participants. |
| 1. Participant knowledge of the interviewer | What did the participants know about the researcher? e.g. *personal goals, reasons for doing the research* | All participants were informed about the researchers had practitioner backgrounds, along with an interest in older people. The reasons for the research being undertaken, including why they had been approached as participants, was made clear to all participants. |
| 1. Interviewer characteristics | What characteristics were reported about the interviewer? e.g. *bias, assumptions, reasons and interests in the research topic?* | Researchers were interested in the experience of making the decision to move from acute hospital to care home. People who were approached to take part in the research were informed about the study teams’ interests in advancing knowledge in this area. |
| Domain 2: Study Design | | |
| Theoretical Framework | | |
| 1. Methodological orientation and theory | What methodological orientation was stated to underpin the study? | Case study research design with inductive thematic analysis. |
| Participant Selection | | |
| 1. Sampling | How were participants selected? *e.g. purposive, convenience, consecutive, snowball* | Purposive sample based on concepts of interest from the research team’s earlier work. |
| 1. Method of approach | How were participants approached? *e.g. face to face, telephone, mail, email* | Face to face approach in inpatient care setting. |
| 1. Sample size | How many participants were in the study? | The sample in this study comprised of the following:  Older People: 6  Significant People: 7  Multidisciplinary Team Members (MDT): 17  Total Sample: 30. |
| 1. Non-participation | How many people refused to participate or dropped out? Reasons? | A total of 4 older people refused to participate in the study due to feeling too unwell.  A total of 8 older people initially approached moved out of the hospital setting between identification and recruitment.  No significant person or MDT member refused to participate. None of the participants dropped out of the study after recruitment. |
| Setting | | |
| 1. Setting of data collection | Where was the data collected? *e.g. home, clinic, work* | All interviews with older persons and multidisciplinary team members took place in meeting rooms in the hospital setting.  Interviews with significant persons were undertaken either in the hospital setting, or their own homes depending on their preference. |
| 1. Presence of non-participants | Was anyone else present besides the participants and researchers? | Nobody else was present at the interviews besides the participant and the researcher. |
| 1. Description of sample | What are the important characteristics of the sample? e.g. demographic data | The sample were older adults who had chosen to move to care home directly from the acute hospital admission. Key demographic data are included in Table 2. |
| Data collection | | |
| 1. Interview guide | Were questions, prompts, guides provided by the authors? Was it pilot tested? | A semi-structured interview guide was developed, piloted and refined in collaboration with the study team and used for all interviews (Appendix 1). |
| 1. Repeat interviews | Were repeat interviews carried out? If yes, how many. | Repeat interviews were not carried out in this study. |
| 1. Audio/visual recording | Did the research use audio or visual recording to collect the data? | All interviews were audio recorded with permission of the participants. Audio recordings were transcribed verbatim by NHS transcription services and checked by the study Principal Investigator (GS) |
| 1. Field notes | Were field notes made during and/or after the interviews? | The interviewing researchers made field notes during the interviews |
| 1. Duration | What was the duration of the interviews? | All interviews were undertaken for a duration of 30-60 minutes. |
| 1. Data saturation | Was data saturation discussed? | This small study was funded for a limited time and therefore data saturation has not been claimed. The data was deemed sufficient to address the research questions. |
| 1. Transcripts returned | Were transcripts returned to the participants for comment and/or correct? | Transcripts were not returned to any of the participants for comment or correction. |
| Domain 3: Analysis and Findings | | |
| Data Analysis | | |
| 1. Number of data coders | How many data coders coded the data? | Two researchers (GS, JKB) coded the transcripts independently within each dataset and then codes, themes and definitions were discussed, reviewed and confirmed among the study team. Codes and themes were refined and individually applied to each dataset followed by the data as a whole. |
| 1. Description of the coding tree | Did authors provide a description of the coding tree? | No description of the coding tree has been provided in this study. |
| 1. Derivation of themes | Were themes identified in advance or derived from the data? | Themes were derived from the data inductively.  Within case analysis ensured perspectives from each stakeholder were fully explored. Cross-case analysis ensured similarities and differences between the cases were made explicit and themes common to all cases could emerge through grouping of individual codes. |
| 1. Software | What software, if applicable, was used to manage the data? | The researchers used NVivo Version 11 to code the data. |
| 1. Participant checking | Did participants provide feedback on the findings? | The participants did not provide feedback on the findings. |
| Reporting | | |
| 1. Quotations presented | Were participant quotations presented to illustrate the themes/findings? Was each quotation identified? *e.g. participant number* | Participant quotations have been presented to illustrate the themes and findings. Pseudonyms were used to identify the older person participants. Significant persons were identified through the relationship type (e.g. daughter, nephew etc.). Multidisciplinary team members were identified through their discipline (e.g. Consultant, Social Worker). |
| 1. Data and findings consistent | Was there consistency between the data presented and the findings? | All findings were derived from the data. Participant quotations were used to demonstrate consistency between the data presented and the findings. |
| 1. Clarity of major themes | Were major themes clearly presented in the findings? | Three major themes were derived from the data and presented in the findings. Each theme includes a short paragraph to introduce a synopsis of the theme. |
| 1. Clarity of minor themes | Is there a description of diverse cases or discussion of minor themes? | Any differences between participant responses are discussed within each theme, with participant quotations used to demonstrate consistency with the description of the findings. |
